# Sex Workers’ Everyday Security in the Netherlands and the Impact of COVID-19

**DOI:** 10.1007/s13178-022-00729-4

**Published:** 2022-05-25

**Authors:** María Inés Cubides Kovacsics, Wáleri Santos, Karin Astrid Siegmann

**Affiliations:** 1 International Institute of Social Studies of Erasmus University Rotterdam (ISS), The Hague, the Netherlands; 2 Rotterdam, the Netherlands; 3 ISS, The Hague, the Netherlands

**Keywords:** Biopolitics, Covid-19, Gender, Insecurities, Legal liminality, The Netherlands, Sex work

## Abstract

**Introduction:**

The COVID-19 pandemic has laid bare and exacerbates the existing insecurities of sex workers. This paper asks: What are sex workers’ everyday experiences of (in)security? And: How has the COVID-19 pandemic influenced these?

**Methods:**

We engage with these questions through collaborative research based on semi-structured interviews carried out in 2019 and 2020 with sex workers in The Hague, the Netherlands.

**Results:**

Revealing a stark mismatch between the insecurities that sex workers’ experience and the concerns enshrined in regulation, our analysis shows that sex workers’ everyday insecurities involve diverse concerns regarding their occupational safety and health, highlighting that work insecurity is more multi-faceted than sexually transmitted infections (STIs). Widespread employment and income insecurities for sex workers are exacerbated for transwomen and male sex workers. Their legal liminality is enabled not only by the opaque legal status of sex work in the Netherlands, but also by the gendering of official regulation. The COVID-19 pandemic made visible how the sexual and gender norms that informally govern sex workers’ working conditions intersect with hierarchies of citizenship, complicating access to COVID-19 support, particularly for migrant sex workers.

**Conclusions:**

Sex work regulation in the Netherlands leaves workers in a limbo—not without obligations and surveillance, yet, without the full guarantee of their labour rights.

**Policy Implications:**

To effectively address sex workers’ insecurities, a shift in regulation from its current biopolitical focus to a labour approach is necessary. Besides, public policy and civil society actors alike need to address the sex industry’s harmful social regulation through hierarchies of gender, sexuality and race.

## Introduction

The COVID-19 pandemic has laid bare and exacerbates communities’ existing insecurities. Clearly, this is the case for sex workers, a highly stigmatised, often criminalised and economically precarious group of workers (de Wildt et al., 2020, p.5; European Parliament, 2021, para. AL). We refer to ‘sex work’ as an alternative to stigmatising language for the labour performed in commercial sex industries (Berg, 2014, p. 693). A few weeks after the first Corona-related national lockdowns in spring 2020, Platt et al. (2020) projected that sex workers’ ‘[…] inability to work, reduced access to health services, and increased isolation are likely to result in poorer health outcomes and increased inequalities, particularly where individuals are largely excluded from formal social protection schemes’ (p. 10). Their prediction implies that the way in which sex workers experience this crisis crucially depends on the legal and social regulation of the sex industry. More specifically, even formally employed sex workers experience a ‘legal limbo’. This context works as a magnifying glass for existing problems, both in terms of making them more visible and by aggravating some of them.

In the Netherlands, sex work is a legal profession and the stated aim of regulation of the sex industry at national and municipal levels is to improve the status and security of sex workers (Tweede Kamer, 2021; Municipality of The Hague, 2019; van Wijk et al., 2014). Despite this conducive framework, sex workers continue to experience different forms of violence (Aidsfonds & PROUD, 2018; Breuer & Intraval, 2018; James & Hamburg, 2020; Pitcher & Wijers, 2014; Verhoeven, 2017). Does this imply that the regulation of the sex industry in the Netherlands is ineffective? Or do understandings of desirable conditions in sex work differ?

Before the onset of the COVID-19 crisis in the Netherlands, these questions motivated a small-scale inquiry into sex workers’ everyday experiences of security in relation to their work. The feminist qualitative study centred around semi-structured interviews with sex workers. While research participants’ gender identities, migratory experiences and work locations varied, they all worked in The Hague, a city where sex work is less visible and less researched compared to Amsterdam’s famous red-light district. Follow-up interviews between June and August 2020 enriched the initial fieldwork conducted in 2019. They sought to understand how the outbreak of COVID-19 and related prevention measures have influenced sex workers’ experiences of (in)security. Based on this, this paper seeks to formulate answers to the questions: What are sex workers’ everyday experiences of (in)security? And: How has the COVID-19 pandemic influenced these?

The paper is based on an innovative methodology that co-creates knowledge with sex workers. Such co-creation of knowledge responds to the feminist call for collaborative research with historically marginalized and stigmatised communities. It is based on the conviction that research for and about women and other marginalized groups is most effectively accomplished through communal rather than hierarchical models of scholarship (Rhoades, 2000, p. 96). More importantly, this orientation is in line with sex workers’ demand not to produce knowledge and design interventions about them without them (Global Network of Sex Work Projects, 2013; International Committee on the Rights of Sex Workers in Europe, 2015).

Thematically, this paper moves away from the dominant frames of deviant behaviour or the transmission of diseases by speaking to an increased interest in sex work as work. Recent special issues on sex work across disciplines, e.g. in Social Sciences (2020), Anti-Trafficking Review (2019), Feminist Economics (2017) and Sociological Perspectives (2016), reflect this scholarly attention. These contributions affirm that, for many sex workers, the entry into sex work represents a key practice to achieve security (Lam & Lepp, 2019, p. 96). This is even more true for trans persons who face severe discrimination, as in the Italian labour market described by D’Ippoliti and Botti (2017). Security achieved by the entry into sex work can then be in terms of securing an income or accessing health services, or even finding a network of support within a familiar community.

In contrast, many scholarly contributions document that—for diverse contexts—regulation whose stated aim is to end sex workers’ exploitation, like anti-trafficking interventions, actually trigger and exacerbate financial insecurity, exploitation and unsafe practices among sex workers (e.g. Hoefinger et al., 2020; Lam & Lepp, 2019; Lutnick, 2019; Parmanand, 2019; Peterson et al., 2019; Villar, 2019). In the context of the 2016 sex purchase ban in France, Calderaro and Giametta (2019) therefore argue that the ‘construction of the “problem of prostitution” should be seen in light of broader political anxieties over sexism in poor neighbourhoods and immigration control, which justify the national priorities of security and public order’ (p. 155) (see also Lerum & Brents, 2016, p. 20).

We apply the concept of everyday security to sex work, an occupation that has been characterised as the ‘ultimate precarious labour’ (Sanders & Hardy, 2013). Crawford and Hutchinson’s (2015) concept of ‘everyday security’ allows us to focus both on the ways in which people experience security projects, strategies and regulations, and on how people create specific practices ‘[…] to govern what they understand and interpret as their own security’ (Crawford & Hutchinson, 2015, p. 1185). Standing (2011, p. 10) underlines that multiple forms of insecurities are characteristic for precarious work. He distinguishes labour-related insecurities with regard to inadequate income-earning opportunities and— associated— insecure income, employment and job insecurity, insufficient protection against risks for health and safety at work, the inability to gain skills and use competencies, as well as the lack of a collective voice in the labour market (Siegmann & Schiphorst, 2016, p. 114).

Based on fresh empirical perspectives from the margins of the Dutch labour market, our paper can contribute to the formulation of more inclusive social policy. The comparison of the 2019 situation with contextual changes resulting from the COVID-19 pandemic is not just a timely topic amidst the ongoing crisis, but also enables the critical interrogation of sex workers’ (in)securities from the perspective of a massive crisis of public health, employment and social protection. This is also relevant for critical discussions around sex work as work in contexts outside Europe where this situation of crisis represents ‘normal’ life for many (e.g. Adebisi et al., 2020; Cabezas, 2004; Parmanand, 2019; Ritterbusch, 2016; Santos et al., 2021). This way, our paper contributes to pressing global debates about inclusion and exclusion in access to social security.

Our paper is structured as follows. The section on ‘Diversifying Conceptualisations of Security in Sex Work: Towards a Labour Approach’ provides a sketch of conceptual resources that enable a deeper discussion of The Hague sex workers’ experiences of (in)security. We describe our collaborative research approach in the ‘Research Methodology’ section. In addition, this section gives an overview over the way in which research participants were invited as well as our methods to generate and analyze data. It is followed by an overview over relevant regulation of the sex industry in the Netherlands and The Hague, in particular (the section on ‘Sex Work in the Netherlands: a Profession in Limbo’). The section ‘Analysing Sex Workers’ Heterogeneous Experiences: the Insecurities of a Profession in Limbo’ introduces sex workers’ diverse experiences of (in)security and how the COVID-19 pandemic has put the latter under a magnifying glass. In the context of sex workers’ ‘legal limbo’ that shifts power from sex workers to business operators, we pinpoint the role of social regulation through gender norms and hierarchies of citizenship for explaining their heterogenous insecurities. In ‘Discussion and Outlook’, the paper concludes with a summary of our findings and an outlook for more inclusive social policy.

## Diversifying Conceptualisations of Security in Sex Work: Towards a Labour Approach

Using the lenses of biopolitics regarding the administration and hierarchies of sexuality and population control, we analyze sex work as a ‘profession in limbo’—legally liminal—that shapes sex workers’ everyday security. We apply a polymorphous approach towards sex work, in which diverse experiences are shaped by intersecting hierarchies of gender, sexuality and citizenship.

Potential insecurities for sex workers are often assessed through the lens of the ‘oppression paradigm’ (Weitzer, 2009). Defendants of this paradigm argue that all sex workers are exposed to violence and exploitation. This paradigm assumes patriarchal gender relations that victimize women and sexual hierarchies that stigmatize remunerated sexual practices (Rubin, 1999 [1984]; Butler, 2009; Weitzer, 2018). This perspective and the underlying hegemonic notions of gender and sexuality are often reflected in policies informed by a criminal law approach to sex work (Heumann et al., 2016), that is further inflected by racialization (e.g. Cabezas, 2004). A criminal law approach focuses on crime, especially on identifying and ‘punishing migration networks and employers of forced labour’ (Heumann et al., 2016, p. 180) and ‘saving’ victims.

The ‘empowerment paradigm’, in contrast, ‘focuses on the ways in which sex work classifies as work, involves human agency, and may be potentially empowering for workers’ (Weitzer, 2009, p. 215). Although this paradigm recognizes sex workers’ voluntary entry into the industry, it falls into the same gender and sexual assumptions as the opposite view. In addition, the patriarchal and heteronormative understandings of the organization of sex work that inform both the oppression and empowerment paradigm lead to the invisibilisation of transwomen and male sex workers that aggravates their legal liminality.

Studies looking at sex work from the perspective of legal liminality (Chun, 2009) are aligned with the alternative ‘polymorphous paradigm’, which argues that: ‘(…) there is a constellation of occupational arrangements, power relations, and worker experiences’ (Weitzer, 2009, p. 215), and these cannot be reduced to either exploitation or empowerment, but rather they are a spectrum of variations and combinations.

Following this approach, we take intersectionality as a key feature of sex workers’ experiences of (in)security. Intersectionality, as a theoretical and methodological perspective, makes it possible to see and explore how multiple identities and structures of oppression and privilege operate in different ways and levels (Winker & Degele, 2011). In that sense, it is possible to understand, for example, how a transgender sex worker from Latin America and a transgender sex worker from the Netherlands experience different structures of oppression (Janssen, 2003), construct their identities in different ways and create different symbolic representations of their experiences and lives. Therefore, a main feature of intersectionality is to acknowledge and analyze how different experiences, caused by different intersections of identities, create new hierarchies and systems of discrimination and oppression. Another important feature of intersectionality is that relations of power are not static, they are continuously changing and they depend on the context, e.g. of the COVID-19 pandemic.

Scholars using the lens of legal liminality to understand sex workers’ experiences argue that their legal status remains ambiguous, like in a limbo, creating a position in which sex work is neither legal nor illegal (Fassi, 2016; Hubbard et al., 2008; Truong et al., 2014). Fassi (2016) states that this legal liminality is contextual, written and performed. This means that the limbo is created by the specific rules where sex workers work, rules which may or may not be enforced (Fassi, 2016, p. 27). This creates an asymmetric situation in which sex workers are not left without obligations and surveillance, but without full labour rights (Fassi, 2016, p. 31).

The idea of a legal limbo makes visible the practical and discursive implications of performing an activity that is neither regulated as work nor punishable as a crime (Fassi, 2016, p. 34). Within this limbo, work-related insecurities offer an operationalization of precarious work (Standing, 2011). The legal limbo also helps us to understand how, without the recognition of sex work as work and a labour approach, it might not be possible to change the existing precarious conditions many sex workers live in. Even more, it is possible to analyze how these conditions are partly produced by this liminal condition (Fassi, 2016, p. 8), understanding how governmentality operates within sex work.

Foucault’s concept of governmentality refers to the mechanisms and technologies, the rationalities, techniques and procedures, by which the state controls specific populations. This is done by re-producing what is considered as ‘normal’ and what is not, and this is usually justified through the idea of security and protection. Foucault also called this biopower (Foucault et al., 2007, p. 1). The mechanisms of security are created to keep specific behaviours and practices, framed as dangerous, ‘within socially and economically acceptable limits […]’ (Foucault et al., 2007, p. 6) in specific contexts. The concept of governmentality helps us to make visible the differences between everyday security versus state-centric notions of security, it allows us to analyze how municipal and national regulation of sex work emerge from and respond to the desire to control populations and normalizing certain behaviours, while sex workers’ everyday experiences and definitions of security point to different objectives and necessities.

## Research Methodology

### Collaborative Research Approach

To position sex workers (or any marginalized group) as the experts of their own lives and experiences is not only an ethical and epistemological issue, but also a political act (Code, 2015; Harding, 1992). As a feminized occupation, sex workers have been constantly excluded (as women have historically) from the debates and decisions that affect their lives (e.g. Lepp & Gerasimov, 2019, pp. 2–3). Starting from their own experiences and opinions is also challenging the notions about ‘who is the expert’ and ‘who we have to be’ (or who we think we are) to decide for others (Dewey et al., 2018).

As a result of this commitment, in the second phase of the study since 2020, we have co-created knowledge with one of the 2019 research participants on board as co-author. Wáleri, a transgender sex worker from Brazil, worked as a sex professional in the Netherlands, in windows and at home, for 4 years. Our collaboration started with the aim to do follow-up qualitative interviews with previous research participants, combined with an ‘open door invitation’ to get involved in the research, more broadly. The process that started this way led to a collaboration in which all three of us joined hands in all stages of the research.

### Research Ethics

Our research was informed by notions of ‘responsible research’ and ‘caring’, developed by Code (2015). Apart from its role in being critical and reflexive towards the research process, caring enables the researcher to constantly reflect on the possible and very real effects of the research.

Based on these ethical commitments, we have tried to take into account the vulnerable situations in which sex workers can be, especially given the polarized visions of debates around sex work. Thus, confidentiality and anonymity were two aspects of fieldwork and data generation that needed to be addressed with every participant.

Finally, it was crucial to remember and reflect on the fact that some participants might not agree on the representations resulting from the research process. To avoid the epistemic injustice involved in such misrepresentation, this implied a constant dialogue which was crucial for the epistemological standpoint of this research and for the collaborative production of knowledge from multiple and diverse perspectives.

### Research Participants

Given the limitations regarding language (since two of the authors do not speak Dutch), the study focuses on English or Spanish-speaking sex workers working in The Hague. Their participation in the study was invited through a combination of chain sampling and maximum variation sampling. The process of chain sampling involves locating information-rich research participants by asking well-situated people for referrals (Patton, 1990, p. 176). It started with gatekeepers from the different non-governmental and self-help organisations—such as the service provider Spot 46 and the sex worker union Liberty—contacting sex workers and inviting them to participate in the project. Moreover, chain sampling was also applied to explore which relevant actors or public institutions beyond participating sex workers could be included in the research.

Maximum variation sampling seeks to identify central themes cutting across a great deal of participant variation (Patton, 1990, p. 172). We translated this in an effort to work with a diverse group of sex workers to identify both differences and commonalities. In practice, both cis- and transgender women working from windows were interviewed. They form the majority of the sex workers working in the licensed sector in The Hague. One cisgender man working from home was also interviewed. As a result, the work location varied, too, as some of these participants combine their job in windows with home-based sex work in the cities where it is allowed. The focus on English and Spanish-speaking sex workers produced diversity regarding research participants’ nationalities and immigration statuses. While two of them were Dutch, the majority originated from different Latin American countries. Holding Spanish, Italian or Portuguese nationalities alongside their native citizenship enabled them to work in the Netherlands as EU citizens.

### Qualitative Interviews

After three exploratory interviews conducted with key informants from sex workers’ and support organisations, one more interview was conducted with an institutional actor, a member of the Security Department—Public Order and Security from the The Hague Municipality. Based on an interview guide that focused on sex workers’ practices and experiences of security, 13 interviews were conducted with sex workers between June and September 2019. Informed consent was obtained from each participant before beginning each interview, and their names were changed for protection, as desired by them. Follow-up conversations with three sex workers and with one member of the support organization Spot 46 were performed to ask specific questions about the interpretation of the results. Finally, to explore the COVID-19-related changes, between June and August 2020, four interviews were conducted with sex workers who participated in the previous study and one with a member of Spot 46.

### Data Analysis

The qualitative data so generated was analyzed with computer support. The software Nvivo and Atlas.ti were used to code all the information with initial open codes and the gradual development of analytical categories. In the process of open coding, the analytical categories of labour rights, responsibility, precarity and working conditions inductively emerged from the interview data. Additionally, the frequency (or ‘groundedness’) of the codes provided pointers regarding the centrality of themes for our analysis. Based on that, the team held regular discussion and analysis meetings during which a conceptual mapping was developed inspired by Ligita et al. (2022). We discussed the interview data by examining the most commonly used codes and their relationships. Some citations selected by topic were discussed to identify differences and similarities and to examine extreme cases. We created networks to visualize the results and analyze the connections between codes. Thus, central themes in relation to the research questions could be identified.

## Sex Work in the Netherlands: a Profession in Limbo

### Regulation of the Sex Industry in the Netherlands

Although sex work is legal in the Netherlands and there is a common public discourse about improving sex workers’ position and creating a safe environment for them to work, Post et al. (2019) point to the paradox that ‘[…] this liberal dream goes hand-in-hand with a growing repression of personal freedom in the Dutch prostitution sector’ (p. 115). The anomaly of sex work being regulated by the Ministry of Justice and Security, rather than by the Ministry of Social Affairs and Employment comes about as the result of the occupation being approached as a security issue, both from the perspective of human and national security. It is seen as a ‘special profession’ with high risks to both sex workers’ health and safety and the potential to harm the public order. As will be detailed below, perversely, this ambiguous framing as a legalized profession that simultaneously poses security risk leaves sex workers in a ‘legal limbo’ (Fassi, 2016) and their rights unprotected (Cubides Kovacsics, 2021).

National and municipal regulation organizes sex work around a licensing system. The term refers to sex businesses such as windows, brothels, private clubs or escort agencies that operate under a license, and where sex workers can provide their services (Rijnink & van Wijk, 2020). An unlicenced sector remains alongside this regulated part of the sex industry. The almost 50% decline in licensed enterprises for sexual services between 2000 and 2014 (van Wijk et al., 2014, p. 38) reflects the growing repression of the sex industry in the Netherlands identified by Post et al. (2019). Most self-employed sex workers working from home have been excluded from the licensing system and the prohibition of home-based sex work in most cities is associated with this lack of regulatory reach and based on the assumption that human trafficking occurs more in the privacy of the home (Post et al., 2019, p. 112; van Stempvoort, 2021, p. 70).

Outshoorn (2012) describes how regulatory discourses frame the two parts of the Dutch sex industry in national terms. The licenced sector is constructed around an ‘[…] ethnically undefined Dutch sex worker who willingly chooses to work in prostitution and is the bearer of civil and social rights’ (p. 237). Moreover, non-EU citizens cannot work legally as sex workers because they cannot ask for a permit themselves (Verhoeven, 2017, p. 371). During the period of our analysis, sex work was the only labour sector for which the Foreign National Employment Act (Art. 8.1) prohibits the issuing of working permits. Sex workers in the unlicenced part of the industry, in contrast, are cast as ‘foreign’ and originating from Eastern Europe and West Africa (Outshoorn, 2012, p. 237). Van Wijk et al., (2010, pp. 202–210) estimate that more than half of the sex workers in the Netherlands are migrants, and a recent report estimates the same for window-based sex workers in The Hague (van Gelder & Veldboom, 2019, p. 19).

Sex workers’ contractual status forms an obstacle to the realization of many rights. While, in theory, they can work as self-employed, as employees or in the so-called opting-in system, in practice, very few sex workers are employees, e.g. of a brothel (Breuer & Intraval, 2018, pp. 6, 19; Rijnink & van Wijk, 2020, p. 22). Under the opting-in arrangement that is common, e.g. in clubs, massage parlours or escort agencies, ‘the operator withholds income tax and VAT on the earnings of sex workers, as in an employment relationship’ (Pitcher & Wijers, 2014, p. 7; van Stempvoort, 2021, p. 68). It is the sex business owner (or operator), rather than the sex worker who decides whether to follow this system or to offer an employment contract (Breuer & Intraval, 2018, p. 4). Some municipalities oblige operators to make use of an opting-in arrangement (van Stempvoort, 2021, p. 69). Window-based sex workers are commonly self-employed (van Stempvoort, 2021, p. 70), as it is the case for sex workers who work from home where it is permitted. The opting-in status implies that ‘[…] sex workers can neither derive any of the rights and benefits of an employee from this arrangement, nor can they derive any of the (tax) benefits of a self-employed worker’ (James & Hamburg, 2020, p. 9). Similar to self-employed sex workers, they have no entitlement to sickness benefits, something that sex workers point out as problematic (Bleeker et al., 2014, pp. 2, 40). Besides, these two dominant employment statuses imply that working conditions in the sex industry, including income and occupational safety and health, do not fall under the ambit of the Dutch Labour Inspectorate (Inspectie SZW [Inspectorate SZW], 2019).

While their ‘legal limbo’ offers them weak protection of their rights at work, sex workers experience pronounced surveillance in the name of the prevention of public health risks as well as of human trafficking (Rijnink & van Wijk, 2020, p. 17). The increasing attention to human trafficking and the framing of the sex industry as its main hub has produced specific strategies and policies that have had a severe impact on sex workers’ lives. For example, policies that prohibit home-based sex work ‘[…] make it difficult to differentiate between human trafficking and what is simply unlicensed home-based sex work. Thus, officially the police are supposed to track down trafficked persons but in practice their efforts mainly impact independent home-workers who run the risk of receiving hefty fines or even eviction’ (James & Hamburg, 2020, p. 14).

### Institutional Set-up of Sex Work in The Hague

Municipalities have been the main shapers of Dutch prostitution policy since the lifting of the ban on brothels (Daalder, 2015, p. 13). Each municipality decides which forms of sex work are allowed, creating specific forms of legal or illegal sex work and associated entitlements (Rijnink & van Wijk, 2020, p. 5). This local regulation is often experienced as ambiguous and unclear by sex workers (Breuer & Intraval, 2018, p. 22).

In The Hague, while street- and home-based sex work is not allowed, the licensed sector is divided into private houses, clubs and the two window areas in the city—Doubletstraat and Geleenstraat. Since 2008, a limit to the number of 85 licensed sex businesses was established employing an estimated 1000–1500 sex workers (Heuts et al., 2012, p. 13; Rijnink & van Wijk, 2020, p. 41). While the exact number of sex workers in unlicensed sectors in The Hague is unknown, in 2012, these were estimated to be at least a few hundred (Heuts et al., 2012, p. 8).

In 2018, the regional public health service (GGD, for its abbreviation in Dutch) reported a total of 845 sex workers who were tested for STIs. This includes 709 women, 115 men and 21 transgender persons, figures which might offer a broader idea of the gender distribution of sex workers in The Hague (van Gelder & Veldboom, 2019, p. 35).

Existing licences have to be renewed annually, for which the formulation of a business plan is required since 2017. The ‘General local regulation’ for the municipality of The Hague (APV for the Dutch acronym), dedicated to public order and safety regulations, stipulates that this plan should indicate the measures that operators take in the field of hygiene, to protect the health, safety and self-determination of sex workers as well as clients and for the prevention of criminal offenses (Municipality of The Hague, 2019, p. 53).

This regulation is enforced through a group of public and private institutions that is chiefly concerned with the guarantee of public health and the prevention of human trafficking. Within the Municipality, three departments are in charge of issues related to sex work: (1) Security (including an urban planning team), (2) GGD and (3) the OCW (Ministry of Education, Culture and Science). The issues they address include policy advice, business supervision, which also involves the identification of human trafficking, spatial planning of sex work locations and, last but not least, health services (Anonymous, interview, 2019; Veldboom, interview, 2019). The GGD is in charge of the latter. In addition, the GGD is in charge of inspecting establishments.

Apart from the Municipality’s departments, the The Hague Economic Intervention Team (HEIT) is in charge of ‘tackling abuses’ in sex work (Municipality The Hague, 2020, p. 10). Although not only related to sex work, the industry is one of their main focus areas. While its stated objective includes both to improve sex workers’ position, counter stigma and to prevent human trafficking (Municipality The Hague, 2020, p. 20), the team has focused on the last goal (Veldboom, interview, 2019).

Apart from these public actors, the service providers Spot 46 and Stichting De Haven as well as the self-help organization Liberty are part of this group of institutions. Being partially publicly funded, the Municipality sees them as part of its security strategy. They offer information and services related to their work, and they support sex workers in their day-to-day needs. This way, these organisations represent sex workers, both in terms of being consulted by the municipality and by local media.

### Sex Workers Falling Through the Cracks of COVID-19 Support

In response to the outbreak of the COVID-19 pandemic in the Netherlands, the provision of direct sexual services was prohibited in the Municipality of The Hague as in the rest of the country from 15 March to 1 July 2020 and again from 15 December 2020. When most other industries, including all other contact-related professions, were allowed to open again in May 2020, sex work was still prohibited (Sekswerkexpertise, 2021). This changed after the lobbying of sex work-related organisations and the creation of a hygiene protocol the industry had to follow to meet certain biosecurity measures (Sekswerkexpertise et al., 2020).

Mirroring the situation of other countries in which the exclusion of sex workers from COVID-19 support packages was widespread (e.g. van Stempvoort, 2021; International Committee on the Rights of Sex Workers in Europe, 2021), despite the payment of taxes and social security contributions by sex workers in the Netherlands, in a situation of crisis, sex workers have fallen through the cracks of social security. Financial support for unemployed sex workers in the form of the Temporary bridging measure for self-employed professionals (TOZO, abbreviated from its Dutch name) was limited to those registered as self-employed in the Chamber of Commerce. Initially, sex workers working within the opting-in system were not entitled to apply to any financial help because as quasi-employees, they cannot register as self-employed (de Wildt et al., 2020, p. 3; van Stempvoort, 2021, p. 68). Yet, self-employed sex workers, too, had difficulties to fulfil the requirements for TOZO, especially the need to hold one of the scarce licences which are commonly held by operators. Even if they did, they could often not access the support (van Stempvoort, 2021, pp. 69–70). TOZO was denied at first to some migrant sex workers regardless of their residence status or to those who had registered for less than a year. In The Hague, income support for persons employed via opting-in was made accessible only four months into the pandemic (Municipal Council The Hague, 2020, pp. 157, 168). As a result, a big group of sex workers working even within the licensed sector were not able to access any financial benefits, and some of them had to continue working to be able to make ends meet.

## Analysing Sex Workers’ Heterogeneous Experiences: the Insecurities of a Profession in Limbo

Sex workers’ experiences of insecurity that come out in our study are more diverse than the concerns enshrined in the regulatory environment. They reflect the intersection of multiple power relations in which their work is embedded.

### ‘The Controls Do Not Serve to Protect Us’—Sex Workers’ Insecurities Amidst ‘Legal Limbo’

Work insecurity as the risk of illness or accidents at work (Standing, 2011, p. 10) is one of the most significant risks that sex workers in The Hague experience. The municipality provides related services that centre around awareness raising about safe sex, free vaccination and STIs testing for sex workers and clients through the GGD and Spot 46 (Municipality of The Hague, 2020, pp. 9–12).

Other risks for sex workers’ occupational safety and health are outside of the municipality’s focus, though. Hygiene, for instance, is an ignored aspect of window-based sex workers’ work security that strongly depends on each window’s operator. Some sex workers express satisfaction about their work conditions in this regard. The operators of their windows take care of cleaning the corridors, rooms and bathrooms and provide them with clean towels and sheets as well as with access to the shower according to sex workers’ needs. This contrasts with the poor physical working conditions that other sex workers experience. For example, Martha, a 50-year-old cisgender woman from the Dominican Republic, refers to poor ventilation and the amount of dust that accumulates in her workspace as a risk factor for her health. This forces her to choose between an unhygienic work environment and the risk of someone unwanted entering her room:

I do not like to work with the door open because I have the right blood to attract anyone, except someone nice ... Then I keep the door closed because I feel better... So that situation affects me a lot... besides the dirt accumulates too much and… I inhale all of it… (2019)

Emotional and psychological health is another important part of sex workers’ occupational safety. George, a home-based 26-year-old male sex worker from Belgium and the Netherlands, points out that the lack of guidance and support when starting to work in the sex industry induces psychological stress:

There is also a great psychological aspect, apart from the material safety conditions, a great psychological influence, and that is something you have to learn, and the difficult thing about sex work is that there is no training on how to do the work. Many times, a person enters this job, they cannot discuss it with their friends, they do not have colleagues with whom to evaluate how they would do it, and there is no education that teaches them the best way to do it. So that makes it very complicated, I think, because you have to learn everything on your own (2019).

George’s and other workers’ experience of being unable to talk about their job with friends or family because of the stigma related to sex work parallels Borg (2017, p. 35) who considers the emotional risks associated with discrimination, societal exclusion, the stress of leading a double life, mental issues, and stigma the greatest risks experienced by sex workers in the Netherlands. These stresses are aggravated by the fact that professional support is often difficult to access. James and Hamburg (2020, p. 8) find that migrant sex workers in the Netherlands who would like to have therapy or support due to their work-related stress experience high barriers, like being placed on long waiting lists, never getting an appointment, or being pressured by the therapist to stop sex work.

Sex workers do not consider existing measures effective for improving their work security. Window operators’ business plan that includes a protocol on health and safety measures is one of the municipality’s key strategies to protect sex workers’ security (Anonymous, interview, 2019). Yet, most sex workers are not aware of this protocol or related official documents. Those who are, do not know its exact contents. Carmen, a 56-year-old cisgender women from Colombia, sees the Municipality as the only body that can hold operators accountable. According to her, sex workers cannot do this directly: Operators usually do not take their demands seriously or workers risk losing their workplace for speaking out, while fearing that the business in question might be closed down, should they approach the municipality (Veldboom, interview, 2019; Pitcher & Wijers, 2014, p. 555). Carmen’s proposal in fact means labour inspection, an enforcement mechanism for decent working conditions from which sex businesses in the Netherlands are exempted. Existing checks by the GGD are announced, giving business managers the time to prepare both the workplace and sex workers (Spot 46, personal communication, August 7, 2019). Miriam, a cisgender window-based sex worker from the Dominican Republic soberly recounts: ‘They let us know when they are going to inspect so we can prepare. We clean and fix’ (2019). Rather than experiencing official checks as effective for their protection, sex workers see existing inspections as forms of surveillance, aimed to identify cases of human trafficking and illegal work:

The controls do not serve to protect us. Only to control that we are with papers in order. The only thing is if you're chuleada [having a pimp], it's the only thing that matters to them. (Wáleri, interview, 2019)

Besides, income insecurity is a key concern for sex workers. A cause of income insecurity that Standing (2011, p. 10) identifies, namely, the lack of protective regulation guaranteeing, e.g. minimum wages or social security, also applies to our research participants. While the window-based sector in The Hague applies a minimum service rate for sexual services, ironically, this rate rather serves as a benchmark and thus makes it more difficult to negotiate a higher amount with clients. Rather than guaranteeing an adequate income, the minimum rate leads to a trade-of between access to clients and access to fair remuneration. Rosa, a 50-year-old cisgender woman from the Dominican Republic, suggests that this results in tensions between sex workers who demand the minimum rate and those who ask for less in order to attract more clients: ‘Many do not want to say it because they get problems [with their peers]… but many of us do it for 20 because we are not doing anything… 20 is better than nothing’ (2019). It is telling that the minimum rate was not something demanded and decided by sex workers themselves but established by operators in Doubletstraat to tackle sex workers’ criticism of the high rental prices they charge (van Wijk & Mascini, 2019, p. 11).

Income insecurity also results from risks being shifted to sex workers. Sex workers’ dependency on operators enables the latter to burden sex workers with costs, e.g. for damage done by aggressive clients. Similarly, if sex workers get sick, they still have to pay the rent of the window unless they notify their absence one day in advance. The possibility to shift risks to window-based sex workers results from the peculiar contractual relation with operators that combines a high degree of dependency with a low degree of social protection (Wagenaar & Altink, 2012, p. 11). This dependency is reflected in an experience shared by Vanessa. She describes how her operator used Vanessa’s dependency on her for access to a legal workspace to avoid tax payments. Like this lady, some operators privilege workers who are willing to pay the rent without asking for a payment receipt, allowing operators to evade taxation:

They wanted to go up 25 ... To 125 euros from October ... I said no. What did the lady do? I work 4 days and she said: ‘one day you don't take the bonus [payment receipt] and you keep paying 100’. (2019)

The role of operators in regulation that sees them as partners in fighting crime contrasts with sex workers’ perception who look at them as their ‘quasi-employer’. Whereas sex workers consider operators important for guaranteeing their occupational safety and health, income and employment security, local authorities foreground their role in the prevention and identification of cases of human trafficking by checking sex workers’ autonomy, criminal activity (van Wijk & Mascini, 2019), and—less so—the sexual health of sex workers. Here, autonomy is understood in terms of working voluntarily, not in the conditions of their work.

Over and above its neglect of many dimensions of sex workers’ material conditions of work, the local regulation of the sex industry aggravates sex worker stigma. Although sexual health-related services are important for sex workers, the focus on them and the neglect of other issues related to occupational safety and health reinforces the stigma that sees sex workers as subjects of unsafe sex practices and bearers of STIs. Through this form of biopower, the municipality legitimates the control and surveillance of sex workers. Legitimated under the discourse of protection and security, this can be understood as a practice of governmentality and normalization. The fact that sex workers’ bodies and sexuality are seen as the target of public health is naturalized, and other bodies are not seen as the target of these interventions, even if all have an impact on public health.

Sex workers’ peculiar contractual relationship with business owners and window operators turns the stated objectives of regulation on its head. Put in place to reduce sex workers’ vulnerability to exploitation and increase their independence, in fact, it makes window-based workers highly dependent on operators’ inclinations in their access to legal workspace. This, in turn, leaves the door wide open for abuses, such as the tax evasion exemplified by Vanessa’s window operator. In sum, the scarcity of legal employment opportunities for sex workers makes what Standing (2011, p. 10) denotes with labour market security—the availability of adequate income-earning opportunities in the labour market—a distant dream for sex workers in The Hague. Window-based workers’ contractual relation with operators does not offer employment security to them, while home-based workers in The Hague are in an even more precarious situation of illegal employment at risk of eviction. The network of institutions enforcing regulation provides the government with control, but neither provides sex workers with labour protection and rights, nor improves their social position. Taken together, this creates a situation aptly characterized as the legal liminality associated with a (disguised) criminal approach to sex work.

### ‘The Operator Takes Advantage Since We Do Not Have Another Place’—Heteronormativity Aggravates Sex Workers’ Insecurities

The way in which sex workers’ work-related insecurities are mediated by heteronormativity challenges conventional assumptions about sex work.

Wage theft as the possibility that clients steal from them, end up not paying for the services or ask to return their money constitutes another great risk that sex workers identify. This happens, for instance, to transgender sex workers who are being asked to return their payment for ‘tricking’ men about their gender identity. Raquel, a 44-year-old transgender woman and window-based sex worker, had to cover the costs of the window that an angry client had smashed. Like her, sex workers are sometimes told by the operator to return the money to avoid problems with clients. Meike, a 50-year-old, Dutch, home-based, transgender sex worker, explains that such risks are lower in home-based work:

[…] if you work in the window it is difficult because they think you are a cis-woman. And if you do it from home it is [arranged] online, then there is no misunderstanding for example about this… so that makes it a little bit safer. (2019)

In the absence of formal rules guaranteeing equitable access, operators’ decisions to whom to rent their windows, too, are shaped by social hierarchies of gender. Few operators rent out windows to transgender sex workers. The latter are therefore hesitant to report poor working conditions or bad treatment. Vanessa, a 50-year-old transgender woman, explains:

They refuse to lease the room and we have to work in the conditions that are there. And the lady takes advantage [of] that since we don't have another place because they don't give us a window, that's what she takes advantage of and thinks: ‘Oh no, these are NOT going to leave’... (2019)

Male sex workers have even fewer opportunities. George explains:

There is only one club in Amsterdam, which is the only one in the entire country, which offers workspaces for men. So, that lack of regularized workspaces, that is a very big problem... Well, that is something that made me feel insecure. (2019)

As a result of such discrimination and exclusion, transgender women and cisgender men are pushed to work illegally since home-based sex work is not permitted in The Hague. The Municipality does not acknowledge this persistent gender-based discrimination and the resulting insecurities, though. Reproducing the gendered underpinnings of the oppression paradigm that focuses on women sex workers as victims of patriarchal oppression and exploitation and invisibilises the existence of male workers who deserve rights and protection, it frames male sex workers’ greater invisibility as a result of their choice (Municipality of The Hague, 2015, p. 34). As the Municipality suspects cases of human trafficking rather than lack of legal workspaces when sex workers work from home, a dedicated team is in charge of scanning pertinent webpages to identify people working from home. The precarious legal status that George and other home-based sex workers experience as a result directly translates into insecure employment and livelihood.

Against the backdrop of the ‘legal limbo’ that affects all sex workers in the Netherlands based on clashes between the formal legal order and ideologies of sexuality (Menjívar & Bibler Coutin, 2014, p. 328), gendered notions of what counts as ‘normal’ or ‘recognizable’ further entrench sex workers’ precarity. This turns the common perception of sex work as risky because of (cisgender) women’s vulnerability under patriarchy upside down. The oppression paradigm perceives insecurities experienced by sex workers as ‘a quintessential expression of patriarchal gender relations’ (Weitzer, 2009, p. 214) that subjugate (cisgender) women engaged in commercial sex to exploitation and violence. In contrast, the aggravated income and employment insecurity that trans and male sex workers in The Hague experience are rooted in operators’ trans- and homophobia.

### Sex Workers’ Magnified Precarity in Pandemic Times

The regulation of the sex industry in The Hague in the first year of the COVID-19 pandemic was ‘a magnifying glass’ (Veldboom, interview, 2020) for the fact that a large group of people work under very precarious conditions in this industry (van Stempvoort, 2021, p. 69).

During the closure of the sex industry between March and July 2020, sex workers’ existing high employment and income insecurity rose massively. For those involved in direct sexual services, securing their livelihood through work was no longer a feasible option. While COVID-19-proof, digital sex work, e.g. for providing webcam sex services, proved to be near impossible due to municipal regulation. Apart from the different skills and technical equipment required (van Stempvoort, 2021, pp. 70–71), for many sex workers this was not an option because they had no private space for it. Even before the pandemic, home-based sex workers risked double punishment. For reasons of privacy, the municipality cannot fine violations of the rule that prohibits home-based sex work in The Hague, but merely notifies the person (Municipality of The Hague, 2020, pp. 20–22). Often, the municipality then conveys to the sex workers’ landlord that sex work is undertaken in their property. Typically, landlords will then discontinue the rental agreement with the sex worker since; otherwise, they themselves risk a heavy fine (Veldboom, interview, 2019). As a consequence, not only will the sex worker be left without a home, but also without a stable income.

Although a large part of the workforce in the Netherlands engaged in mandatory work from home during the COVID-19 pandemic and working online was not officially prohibited as it does not imply physical contact, the police kept persecuting sex workers who advertised online, arguing they could not work from home. They even threatened to fine sex workers who kept their ads online (Veldboom, interview, 2020; SekswerkExpertise, 2020, p. 11). But sex workers wanted to keep them, even if they were not working in person or not at all, to stay in contact with their clients or to work digitally. Different organisations pointed out that no other service provider was asked to remove its advertisement during lockdown and protested this discriminatory treatment of sex workers. The discrimination regarding advertisements further hampered outreach to possible clients and has provided a window of opportunity for abolitionists. Following efforts of the US government to curb the use of internet facilitation for sexual services (Weitzer, 2019, pp. 407–408), this is reflected in a series of parliamentary initiatives to restrict the use of online platforms for sex work (Tweede Kamer, 2021).

Once window-based sex businesses re-opened, sex workers’ work insecurity increased when operators prioritized their earnings over sex workers’ occupational safety and health. Some sex workers started working immediately when sex businesses were allowed to re-open and sex services were permitted again in July 2020. Others, like Adriana, a cisgender woman who has been in the industry for more than 20 years, decided to wait a little longer to gauge whether the return to work would feel safe. The experience of those who went back to work were diverse. Some workers encountered safe conditions, with operators following the recommendations by providing antibacterial gel, several towels and sheets to change after each client, cleaning alcohol, other cleaning products, making a thermometer available and providing sex workers with face masks. Others did not have support from the operators, who even told them that their safety was their own responsibility. Adriana illustrates this based on a neighbouring worker’s experience:

So I have contact with one girl there, that she works next to my room, and she says nothing changes. Only you get one-liter alcohol and one spray to clean around the bed. But the towel… you work with the same towel all day. I said: 'you [are] crazy'. (2020)

As explained above, sex workers who wanted to shift their work to their home for a more effective guarantee of hygienic and safe conditions were punished with illegality. On top of these heightened health risks, for some of the sex workers who returned to work, income insecurity increased, too. With both sex workers and clients concerned about a COVID-19 infection, overall, the number of clients had dwindled, further aggravated by sex workers limiting themselves to regular clients (see also de Wildt et al., 2020).

Over and above these increased insecurities, the pandemic also revealed how hierarchies of citizenship aggravate sex workers’ conditions. Migrant sex workers had diverse experiences with the public financial support in terms of accessing information about it, and the ease or difficulty as well as the success of the application. Carmen and Wáleri’s experiences exemplify some of these differences:

For me it was easy, I passed it and I had no problem… For other girls it was not like that… for some just until now [July 30] help is coming (Carmen 2020).

On March 22 when it closed, we passed the papers and mine came out on June 17, and it didn't come out complete, it came out 2 months and I still have one missing (...) (Wáleri 2020).

This diversity of experiences can be explained by the measures’ implicit bias against applicants with poorer language skills and insecure immigration status. Some sex workers had trouble understanding the information provided on COVID-19-related support because the language and the technicality of the required information formed a barrier. The greater complexity of an alternative application for social security (*bijstand*) made this form of support even less accessible for non-Dutch natives. More generally, our research participants pointed out that not being Dutch makes it more difficult, for example, to access public services, register at the Chamber of Commerce, pay taxes, communicate with public servants, etc. Earlier, Borg (2017, p. 47) flagged that migrant sex workers’ difficulties to navigate the system is a source of stress and insecurity in itself. For some sex workers, having Dutch speaking networks is the only reason they now have access to their rights and know how to navigate the system. Confirming Borg (2017, p. 33) who identifies language proficiency as one of the most crucial empowering factors in sex work, related to the migratory status and nationality, in our study, too, it emerges as an enabling factor for sex workers to realise their rights. Over and above these skills, for persons who have resided in the Netherlands for less than 5 years, submitting an application for *bijstand* may have negative formal consequences for their residence permit, making it ‘[…] conceivable that this barrier excludes a substantial group from emergency aid’ (van Stempvoort, 2021, p. 68).

The lack of sex workers’ social security that COVID-19 put under the magnifying glass often exacerbated existing dependencies. Once savings were depleted, some sex workers asked operators or clients for loans to make needs meet (Wáleri, interview, 2020; see also de Wildt et al., 2020). The consequence of this is that they would have to work without or for little pay for some time to return the money after the lockdown. While financial dependency is considered an indicator of exploitation (Hoge Raad der Nederlanden, 2019), such perverse consequences of the failure of the state to guarantee sex workers’ social security do not figure in the often scandalistic public discourses about sex work in the Netherlands (see, e.g. Outshoorn, 2012, pp. 237–239).

## Discussion and Outlook

Our analysis brings to the fore a great diversity of sex workers’ needs in terms of their security, needs that remain unaddressed in the governance of the sex industry in the Netherlands. Sex workers’ everyday insecurities revolve around different concerns regarding their occupational safety and health, highlighting that work insecurity is more multi-faceted than STIs alone. The exceptionalism of sex work in Dutch regulation rooted in sexual hierarchies that look at commercial sexual services with suspicion turns sex work into a ‘profession in limbo’—legalized, but unprotected. This context that leaves them with weak rights and their enforcement by the state strengthens the position of operators. Given their resulting dependency from such ‘quasi-employers’, sex workers’ concerns about their health, hygiene and safety are intertwined with employment and income insecurities. It disciplines them not to speak out about degraded working conditions for fear of losing access to a legal workspace. Turning dominant discourses on sex workers’ insecurities topsy-turvy that foreground cisgender women sex workers’ vulnerabilities, we explain part of the diversity of sex workers’ experiences with gender hierarchies that exacerbate employment and income insecurities for transwomen and male sex workers. Acting as a magnifying glass for insecurities that sex workers have faced for long, the COVID-19 pandemic and the containment measures it brought made visible how the sexual and gender norms that informally govern sex workers’ working conditions intersect with hierarchies of citizenship, complicating access to COVID-19 support for migrant sex workers, in general, and even more so for those without an EU-nationality.

### Out of the Limbo Through a Labour Approach

To effectively address the insecurities that sex workers experience and fear, a shift in regulation from its current biopolitical focus to a labour approach is necessary. Verhoeven (2017, p. 371) describes a mix of a criminal and administrative law approach in the Netherlands that seek to combine the fight against human trafficking with better control and regulation. The findings presented above raise question marks about whether this two-pronged approach serves sex workers’ security. Similar to the experiences from other contexts reported in the introduction, they demonstrate that the focus on human trafficking in fact increases sex workers’ everyday insecurities, especially by conceptualizing the private space as prone to trafficking and by illegalizing home-based sex workers based on such an understanding. While sex workers’ ‘autonomy’ has become a litmus test for Dutch law enforcement to distinguish between voluntary and forced sex work, focusing on the role of pimps, this ignores sex workers’ dependency from operators and the degrading working conditions that may result from it.

Similarly, the narrow focus of municipal regulation on sex workers’ health as STI prevention reproduces misleading imaginaries of sex workers as vectors of disease who pose a risk to public health (e.g. Hubbard et al., 2008, pp. 137–138; Vanwesenbeeck, 2001, p. 245) rather than addressing the wider range of work insecurities that sex workers face. Taken together, sex work governance seems to be motivated by an urge to control populations rather than by their recognition as an occupational group that deserves rights and respect. Heumann et al., (2016, pp. 181–182) label this alternative as a labour approach to sex work. They argue that it starts from the recognition that sex work is work like any other, respects sex workers’ knowledge and demands and therefore involves their representatives in relevant policy debates. Being work like any other has been translated as a demand for the decriminalization of sex work, implying that ‘[…] no particular laws other than regular employment laws address commercial sex’ (Vanwesenbeeck, 2017, p. 1631; also see Soa Aids Nederland PROUD, & PITCH, 2018). It is encouraging that a proposal in support of this demand was adopted by the Municipal Council in December 2021 (Municipal Council The Hague, 2021). Shifting the supervisory authority of employment in the sex industry to the Ministry of Social Affairs and Employment as also demanded in recent parliamentary debates (Tweede Kamer, 2021) and broadening the mandate of the Dutch Labour Inspectorate to monitor working conditions in the sex industry beyond suspected cases of human trafficking would be logical consequences of such decriminalization.

Taken seriously, an important implication of a labour approach to sex work in The Hague would be to critically interrogate the mapping of public regulation and sex workers’ security. Municipal regulation that limits sex workers’ employment to public spaces in windows, clubs etc. and prohibits home-based work is justified in the name of protecting sex workers by making them visible. We show that this logic in fact produces more precarity for some sex workers while the privacy of their home may enable a better screening of clients and a more effective guarantee of their occupational safety and health. Besides, invisibility can be a strategy to resist the impact of the stigma related to sex work by reducing the emotional stress that comes with public discrimination, stigma and violence (Ham & Gerard, 2014, p. 307; Weitzer, 2009, pp. 221–222).

Decriminalization is not sufficient, though. Public policy and civil society actors alike need to address the social regulation of the sex industry through gender and sexual hierarchies that underpin sex worker stigma as well as migrants’ discrimination which have come out as powerful mediators of sex workers’ insecurities. Other studies have shown how policies that do not take sex worker stigma into account in fact reproduce and exacerbate it (Foley, 2017; Pitcher, 2019). In our study, a heteronormative understanding of the labour process in sex work—where men are looking for sex sold by cisgender women and where gay men should not be visible—in combination with the role of operators as powerful mediators of remunerative employment in the sex industry complicates transwomen and male sex workers’ access to remunerative and safe workspaces. Similarly, hierarchies of citizenship pose hurdles to the effective guarantee of their rights to migrant sex workers even in the licensed sex industry. The wide-spread public portrayal of migrant sex workers as victims of human trafficking (Outshoorn, 2001, p. 485) obscures their actual needs and demands.

Starting from the views of sex workers who participated in this study, a consistent application of a labour approach would include specific rules that prevent gender-based discrimination in accessing workplaces, and concrete strategies to promote the rental of spaces for all genders and the opening of new places for men. For guaranteeing employment security, the number of licenses should be unfrozen in order to offer more job opportunities that match the demand for it. By recognizing other forms of ‘normality’, understanding sex work as a profession open to all genders, the governance around it would not discriminate trans and male workers. In this sense, legal and social regulation will not centre around gender, sexuality and citizenship, but around labour rights.

To ensure that all people in the sex industry can choose the safest form of work according to their particular needs, and to increase income and employment security, independent workers (including those working from home, in other private places, or virtually) must also be entitled to social security, with clear and accessible information (de Wildt et al., 2020, p. 27). Along the same lines, the best way to protect non-EU migrants is precisely by granting them work permits so that they can exercise sex work legally with the protections and rights of any worker, because it is actually the illegal status they are forced to work in—produced by the binary of the migrant victim of trafficking versus the empowered citizen—what makes them vulnerable and unprotected (van Stempvoort, 2021, p. 72).

With the aim of supervising working conditions, an anonymous system of complaints should be created, without this being a risk of business closure, in order to avoid that workers have to choose between not losing more spaces and good working conditions. Likewise, it is necessary to create more concrete, clearer and enforced guidelines on health and safety at work, which should be built by consulting sex workers about work security needs. These guidelines should be socialized with the workers so that they know how to demand their rights.

Regarding sex workers’ training and emotional wellbeing, spaces should be created (de Wildt et al., 2020, p. 24; Breuer and Intraval, 2018, p. 72) for sex workers to meet other sex workers, create support networks, learn about their rights and how to access them and share security strategies in their work. Access to psychological and emotional therapy should be also guaranteed, without discrimination or pressure about exiting the industry.

The binary understandings within sex work that separate it from regular labour norms do not serve to protect sex workers. Instead, regulation should take sex workers’ experiences and diverse needs into account and acknowledge the benefit of different work alternatives for accessing labour rights and protection. Through the lens of the polymorphous paradigm and understanding intersectionality as a key feature of sex workers’ experiences of (in)security, sex work regulation in the Netherlands is revealed as a limbo that does not leave workers without obligations and surveillance, but without full labour rights. If sex work is seriously treated as any other work by state regulation, sex workers’ position will improve, and their everyday insecurities will decrease: it would stop being a Profession in Limbo.
